# How to Make Nothing Out of Something: Analyses of the Impact of Study Sampling and Statistical Interpretation in Misleading Meta-Analytic Conclusions

**DOI:** 10.3389/fpsyg.2016.01639

**Published:** 2016-10-25

**Authors:** Michael R. Cunningham, Roy F. Baumeister

**Affiliations:** ^1^Department of Communication, University of Louisville, LouisvilleKY, USA; ^2^Department of Psychology, University of Queensland, BrisbaneQLD, Australia; ^3^Department of Psychology, Florida State University, TallahasseeFL, USA

**Keywords:** self-control, ego depletion, strength depletion, meta-analysis as topic, Precision Effects Test, trim and fill, Test for Excess Significance, Funnel Plot Asymmetry Test

## Abstract

The limited resource model states that self-control is governed by a relatively finite set of inner resources on which people draw when exerting willpower. Once self-control resources have been used up or depleted, they are less available for other self-control tasks, leading to a decrement in subsequent self-control success. The depletion effect has been studied for over 20 years, tested or extended in more than 600 studies, and supported in an independent meta-analysis (Hagger et al., 2010). Meta-analyses are supposed to reduce bias in literature reviews. Carter et al.’s (2015) meta-analysis, by contrast, included a series of questionable decisions involving sampling, methods, and data analysis. We provide quantitative analyses of key sampling issues: exclusion of many of the best depletion studies based on idiosyncratic criteria and the emphasis on mini meta-analyses with low statistical power as opposed to the overall depletion effect. We discuss two key methodological issues: failure to code for research quality, and the quantitative impact of weak studies by novice researchers. We discuss two key data analysis issues: questionable interpretation of the results of trim and fill and Funnel Plot Asymmetry test procedures, and the use and misinterpretation of the untested Precision Effect Test and Precision Effect Estimate with Standard Error (PEESE) procedures. Despite these serious problems, the Carter et al. (2015) meta-analysis results actually indicate that there is a real depletion effect – contrary to their title.

## Introduction

The strength depletion model of self-control (Baumeister et al., 1998) proposes that people have a limited capacity for self-regulation, and this capacity fluctuates due to circumstances and resources. After resisting some temptations or stifling some emotions, the capacity may be reduced. Laboratory experiments have tested this idea by showing that after people exert self-regulation on one task or in one context, their performance on a second, seemingly unrelated self-control task is impaired. A large volume of published studies has demonstrated this pattern with many different procedures and laboratories, as confirmed by meta-analysis (Hagger et al., 2010).

A contrary conclusion was recently asserted by Carter et al. (2015). They conducted a meta-analysis on a small portion of the literature. The second part of Carter et al.’s (2015) title makes the sweeping assertion: “Self-Control Does Not Seem to Rely on a Limited Resource.” That claim is consistent with their previously avowed skepticism about ego depletion (Carter and McCullough, 2013a,b, 2014). In this article, we elucidate why their conclusion seems wrong.

## Nothing or Something?

Carter et al.’s (2015) conclusion that there is no evidence for an ego depletion effect is sharply at odds with the meta-analysis by Hagger et al. (2010). Carter et al. (2015) criticize Hagger et al. (2010) for including only published studies. Published studies have the virtue of the methodological and interpretational quality control that comes from peer review, which can be sorely lacking in unpublished reports. But, the preference for significant results in the publication process can exert a bias in a meta-analytic sample (Greenwald, 1975; Rosenthal, 1979). As we shall note, however, Carter et al. (2015) discarded most of the relevant published literature and replaced it with other work, emphasizing a set of unpublished studies from a small group of investigators. One can readily assume that such a practice will diminish an effect. Even so, what Carter et al. (2015) found was not substantially different. Hagger et al.’s (2010) meta-analysis of *k* = 198 published experiments with 10,782 participants concluded that: “the depletion effect is real, robust to experimental context, and, in terms of a standardized mean difference (i.e., Cohen’s *d*), of medium-to-large magnitude: *d* = 0.62” (CI_0.95_ [0.57–0.67], p. 508). Carter et al.’s (2015) broadest analysis yielded a supportive *g* = 0.43 (CI_0.95_ [0.34–0.52]) in favor of a medium-sized ego depletion effect in their sample of studies. Hedge’s g is akin to Cohen’s *d*, and corrects for small sample effects, so it is appropriate to compare the numbers (Hedges and Olkin, 1985). Thus, while the smaller Carter et al. (2015) sample produced an effect size that was less than the lower bounds of the Hagger et al. (2010) confidence interval, the two very different meta-analyses yielded fairly comparable effect sizes that were consistent with the strength depletion model.

The Hagger et al. (2010) estimate may be high because of publication bias and the Carter et al. (2015) estimate may be low because of their favoring of studies with methodological limitations, as will be described below. Nonetheless, both meta-analyses point to conclusions that are contrary to the Carter et al. (2015) subtitle, namely that there is evidence in favor of the self-control depletion effect.

Moreover, the directionality of the findings aggregated by Carter et al. (2015) is inconsistent with their apparent acceptance of the null hypothesis. If the true effect of ego depletion procedures were zero, then all the significant findings represent capitalizing on chance. Chance works both ways, so about half the time ego depletion should produce better performance, half the time worse (apart from some no-difference findings; Hagger and Chatzisarantis, 2014). Carter et al. (2015) found that in 76% of the studies having a *g* larger than 0.10, the direction was consistent with ego depletion — and a tiny 10% in the other direction. The rest were the essentially no-difference effects, -0.10 < g < +0.10. These tallies include non-significant findings. Carter et al. (2015) did not report how many of these were significant, but it seems likely that most if not all of the reverse-direction findings were non-significant, and reasonably attributable to chance.

As far as we can ascertain, the published literature contains hardly any findings that indicate significant improvement in self-regulatory performance following an ego depletion procedure. Such findings would presumably get high priority for publication. The handful that have been published (e.g., Apfelbaum and Sommers, 2009; DeWall et al., 2011; Carter and McCullough, 2013a; Tuk et al., 2015) reflect highly unusual, specifically designed circumstances. For example, DeWall et al. (2011) found that leaders often disdained low-level work, but when they were depleted, they ceased to assess whether tasks were suitable for them and simply did their best on everything, resulting in an improvement compared to non-depleted leaders. This is not a finding that contradicts ego depletion, but rather it reflects another form that ego depletion can take, namely skimping on non-essential cognitive work (pre-performance assessment).

The substantial absence of published evidence for significant improvements in self-regulatory performance after ego depletion stands in sharp contrast to the 100s of findings of significant decrements. It renders highly implausible the conclusion that the true effect is zero.

We hasten to add that the volume of supportive findings does not prove that the strength or limited model is correct. Our point is simply that there is a genuine phenomenon. Competing models have been proposed (e.g., Inzlicht and Schmeichel, 2012). There seems ample room to debate exactly what the process is that produces these effects. But debating whether there is any effect at all seems unwarranted at this point.

## Excluding Published Evidence

How, then, did Carter et al. (2015) get from the literature’s robust overall support for ego depletion to their conclusion that there is not an effect? Explication of Carter et al.’s (2015) questionable decisions can provide instructive lessons for future investigators, meta-analysts, journal reviewers and authors.

We had serious concerns about the study sampling procedures used by Carter et al. (2015). As noted above, Carter et al. (2015) excluded most of the published literature. They said that they found 620 experiments on depletion, yet their largest meta-analysis included only 116 of these, with 118 effects. Although the exclusion criteria were described, how they got from 620 to 118 effects is not completely transparent. Ideally, two or more unbiased raters should each make exclusion decisions independently, with high inter-rater reliability, but that was not reported by Carter et al. (2015). Only 28 of the 198 experiments included in Hagger et al.’s (2010) meta-analysis survived Carter et al.’s (2015) idiosyncratic sampling to merit inclusion. The high rate of exclusion was explained based on Carter et al.’s (2015) intention to do mini-meta-analyses on specific ego-depletion procedures, so only studies that fit into their eight narrow procedural categories were included. Yet, that justification is questionable because their analyses never focused on the suitability of each procedures as an operationalization of depletion, as will be described below, whereas they offered a sweeping negative conclusion about the depletion effect as a whole, including in their title.

Carter et al.’s (2015) stated objections about much of the literature are unconvincing. Take, for example, their complaint about findings showing that both more (Janssen et al., 2008) and less prosociality (e.g., DeWall et al., 2008) can be taken as signs of ego depletion. Depleted subjects often comply more than control participants with requests for help (Janssen et al., 2008; Fennis et al., 2009). Yet selfish motivations, which mitigate against helping, also were found to increase among depleted persons (DeWall et al., 2008). Although the outcomes point in different directions, the underlying mechanism of failing to inhibit impulses is the same. Indeed, one research team demonstrated both effects, which systematically varied overriding impulses or incipient urges, so that donating more or less can both reflect failure to restrain impulses. They stimulated impulses to be generous by exposing participants to prosocial situational cues, and found that those cues elicited more prosocial action as a function of the situation structure (Halali et al., 2013). Such work illuminates process and builds theory. Ignoring viable findings underestimates the empirical support for the theory that is supposedly being evaluated.

## The Sampling of Unpublished Reports

Carter et al. (2015) stated that they went to some lengths to locate unpublished reports. Published experiments comprised 66.3% (411/620) of their population of studies and unpublished studies comprised 33.7%. (209/620), but the true proportion may be different. The meta-analysts counted a study as published if it was “in peer-reviewed journals, in press, under review, or being sent in for review” (p. 800). Clearly, that procedure mistakenly counts some unpublished studies as published (cf. Cooper, 2009; Grijalva et al., 2015). In the final sample decided by their inclusion criteria, however, published effects were underrepresented by 8.7%, comprising 57.6% (68/118) of the final sample, whereas unpublished effects were over-represented at more than 42.2% (50/118).

The overrepresentation of unpublished tests of depletion in the Carter et al. (2015) meta-analysis sample compared to the population of studies was significant (*z* = 1.79, *p* < 0.04, one tail). Regrettably, Carter et al. (2015) did not report the magnitude of the effect sizes for all 620 studies. They did, however, report that “published” studies in their small and idiosyncratic sample tended to have larger effects than unpublished studies (*b* = 0.18, *p* < 0.06, Table 4). Thus, because unpublished studies tended to have smaller effect sizes, including them disproportionately may tend to bias the meta-analytic outcome.

Even so, the unpublished studies were not randomly distributed, as one would expect if they consisted of reports of competent studies testing a false hypothesis (Hagger and Chatzisarantis, 2014). Inspection of the effect sizes in Carter et al.’s (2015) Table 1, and counting only the unpublished studies, reveals that 75.5% are in the predicted positive direction and only 24.5% (12/49) of the unpublished studies are in the contrary direction (and most of the latter are non-significant). The difference in direction of effects is highly significant (*z* = 3.43, *p* < 0.001), and suggests that the unpublished literature does not consist of a large number of counter-theoretical outcomes, as would be expected if the self-regulation depletion model was spurious. Thus, the aggregate of unpublished studies from the file drawers adds up to weak evidence in favor ego depletion, rather than indicating a large body of genuinely null or contrary findings. This is what one would expect if many unpublished studies with weak or null results failed because of inappropriate calibration, low power, and other missteps by the experimenters.

## Methodological Quality and Null Findings

The authority and insight of meta-analysis come from combining results from as many studies as possible and coding them by methodological quality and characteristics. For example, Tannenbaum et al. (2015) meta-analysis of 127 studies of fear appeals coded for a dozen moderators. Grijalva et al.’s (2015) meta-analysis of 355 studies of narcissism separately coded for five publication types and five sample types. Meta-analyses that fail to include such codes, or that omit much of the literature, lose this advantage. Research synthesists also recommend that each predictor or outcome measure should be examined for the degree to which it operationalizes the constructs of interest (e.g., Cooper, 2009). Regrettably, Carter et al. (2015) did not code studies based on the quality of the methodology or determine if there were appropriately operationalized constructs that provided a genuine test of the depletion hypothesis.

The recent crisis in social psychology over replicability of findings is partly based on the assumption that the same procedures should yield the same effects on anyone, anywhere, and anytime. We think this assumption is generally false. For many psychological phenomena, manipulations and measures often need to be calibrated to the participant population being tested. For example, a bowl of delicious chocolates may not have the same motivational impact on a research participant who has just had a big lunch compared to a dieting participant who skipped lunch (Nordgren et al., 2009).

This simple truth is often overlooked. Carter et al. (2015) criticized the magnitude of effects obtained with anagrams— but anagrams can be so simple that everyone can solve them, or so difficult that no one can. Crucially, the appropriate “sweet spot” level of difficulty is different for different groups, as one of us can attest based on having collected data both at a very selective Ivy League university and a not-very-selective state university. Quite simply, a participant who is already unable to solve an anagram such as ELOTME^1^ will not show any decline in performance due to ego depletion, even if ego depletion makes him or her less able to solve anagrams. Null results based on anagrams that are too hard or too easy for a local population are not failures to replicate (though Carter et al., 2015 would count them as such). Rather, they are failures to properly operationalize and test the hypothesis.

In general, an effective ego depletion operationalization will include an initial task that is sufficiently mentally fatiguing that it degrades cognitive resources, but is not so personally relevant, interesting or challenging that it activates energy reserves. A second task indicative of self-regulation should be presented promptly, before the participant recovers, without a rest period or other procedures, such as several self-report measures. The second task must be based on a strong habit that must be over-ridden through impulse control, such as the Stroop test, refraining from snacking, or solving scrambled words. Thus, the outcome task must require some self-regulatory effort, but also affords the opportunity for participants to slack off without self-awareness or the loss of external incentives, including experimenter approval. A skeptical, distracted, or disinterested (or just absent) experimenter may alter the motivational dynamics of the situation. Pilot-testing with manipulation checks and thorough debriefing should be mandatory before a full study is executed. Purely cognitive tasks that do not involve a conflict with a habitual impulse may be ineffective methods for studying ego depletion (Inzlicht et al., 2016), especially computer-administered measures of executive functioning (Duckworth and Kern, 2016). Unfortunately, this includes the recent registered replication report involving a modification of the Sripada et al. (2014) procedure, whose outcome task simply asked participants to press a button to indicate whether each word has an “e” that is not adjacent to another vowel (Baumeister and Vohs, 2016).

Further illustrating the adverse impact of questionable procedures on ego depletion results is the Carter and McCullough (2013a) study. That extension study purported to assess ego depletion at the end of six disparate activities, including two waiting periods and consumption of sweets in some conditions. The outcome measure was the operation span test of working memory, in which participants were asked to memorize 15 sets of words in blocks of two to five words. The need for impulse control in that task is unclear. The study also included the decision to “restrict data collection to a single semester (p. 3),” thereby reducing statistical power. All of these methodological choices made the results ambiguous, at best. Yet, that study was included in the Carter et al. (2015) meta-analysis without any indication of its weaknesses or complexities. Other studies included in that meta-analysis sample may have had similar issues, which should have been disclosed through proper coding and analysis of methodological moderators.

Meta-analysts struggle with how to deal with null findings, and with good reason. Some null findings indicate highly competent work. These should count as strong evidence against the hypothesis. Others indicate less informative or competent work, including failures to match the procedures to the population, an inappropriate outcome measure, inconsistent experimenter behavior or other extraneous factors. These should not count, or not count as much, as relevant evidence. Research synthesists who disproportionately sample unvetted studies and do not code for methodological quality risk invalid meta-analytic results that mislead the field.

## The Impact of New Investigators

One mixed blessing of a research area that is growing in popularity is the interest of new investigators. Some new investigators offer fresh perspectives and contribute insightful extensions or clarifications of established phenomena. Others wish only to bask in the reflected glory of a hot topic by executing an apparently quick and easy replication project. The latter researchers may try to perform replication studies by copying procedures verbatim from other labs, rather than calibrating them to their participant population. Or, they may use the smallest sample size to be found among the published studies and lack adequate statistical power for the reliability of their execution of the study. A sample size that is appropriate for a carefully run study may be insufficient if the study is run hastily and with errors in an effort to meet graduate program or personal deadlines. Although, an underpowered study can produce a spurious over-estimate of an effect (Button et al., 2013), poor research design and execution, including inadequate power, tend to produce null effects.

Anyone who has served on university thesis committees can attest to the variability in the competence and commitment of new researchers. Nonetheless, a graduate committee may decide to accept weak and unsuccessful replication studies to fulfill degree requirements if the student appears to have learned from the mistakes. There often is little recognition that an error-laden student thesis or conference report may end up in a meta-analysis.

One consequence of a shift from rigorous pioneering work to imprecise student follow-ups would be a general decline in effect size over time. Carter et al. (2015) provide data that allow us to determine that this may be happening with ego depletion. The studies used by Carter et al. (2015) with later reporting dates have significantly lower effect sizes (*r* = -0.33, *p* < 0.0001) and are less likely to be published (*r* = -0.30, *p* < 0.001) than studies from earlier years^2^. This is consistent with our expectations that all research is not created equally and that replication studies, especially by novice investigators, may not be executed with same meticulousness as the original research. Our analyses also are consistent with a recent study that found that replications run by high-expertise teams, with 10 or more publications, produced effect sizes that were nearly twice as large as those obtained by low-expertise teams, in part due to wiser choices of the specific method to replicate (Bench et al., 2017).

Unpublished studies are (by definition) harder for a meta-analyst to find than published ones, and so samples of unpublished studies are likely to be haphazard. Carter et al. (2015) relied heavily on a small group of new researchers who produced many unpublished and non-significant findings. The 50 unpublished effects in Carter et al.’s (2015) Table 1 were attributed to just 20 first authors or teams, who were linked to an average of 2.50 studies each. In contrast, the 68 published effects were produced by 42 different first authors or discrete research teams, with each contributing an average of 1.62 studies each, which is a significant difference from the unpublished studies, χ^2^(61) = 114.72, *p* < 0.001^3^. In addition, nearly two-thirds of the unpublished effects (32/50) were from theses or dissertations produced by just *10* graduate students, averaging 3.2 studies each. One student was associated with *10* and another student with *7* unpublished studies in the dataset, which seems disproportionate. This raises the possibility that the results of a small number of unpublished graduate students were given substantially more weight per person in the Carter et al. (2015) meta-analysis compared to the work of more successful investigators.

While the 64% rate of graduate student authorship of the unpublished Carter et al. (2015) effects seems high, comparable figures for the published studies are lacking. Unfortunately, the student status of authors is not disclosed in most published reports. It is noteworthy, however, that the unpublished effects, which included conference presentations, had a mean of 1.48 authors compared to 2.96 authors for the published findings [*F*(1,116) = 46.27, *p* < 0.0001]. The greater number of authors of published than unpublished studies raises the possibility that more care and professional attention were devoted to successful than unsuccessful research.

Novice investigators’ shortcomings, such as failure to properly operationalize and pilot-test procedures, can have a disproportionate impact on meta-analytic results. There was a significant relation of source of study (unpublished thesis/dissertation; other unpublished report; published report) to sample size in Carter et al. (2015) [*F*(2,117) = 5.28, *p* = 0.0005]. Of particular interest, the *k* = 32 unpublished theses and dissertations, which can be conclusively attributed to new investigators, had significantly smaller sample sizes than the *k* = 68 published studies [*n* = 41.88 vs. 59.37, *t*(98) = 2.63, *p* = 0.01], producing lower statistical power and less likelihood of a significant effect. Those unpublished theses and dissertations also had higher variance of the effect size estimates than the published studies [*v* = 0.103 vs. 0.086, *t*(98) = 1.84, *p* = 0.03, one-tail]. In light of such quality control questions in the unpublished literature, we sympathize with Hagger et al.’s (2010) decision to focus on published studies in their meta-analysis.

These issues should have been addressed by Carter et al. (2015). In a review of meta-analyses, Coyne et al. (2011) found that dissertations typically were statistically underpowered and had other methodological deficiencies. They concluded: “The uncritical inclusion of unpublished dissertation studies in meta-analyses should be discouraged. A more judicious decision would be to base inclusion in meta-analyses on study quality or, at a minimum, to present results for high- and low quality studies separately (p. 225).” The failure of Carter et al. (2015) to follow this reasonable recommendation likely contributed to their more negative assessment of the self-control literature compared to Hagger et al. (2010).

The professional literature has an intentional “quality bias” in favor of studies with theoretical and methodological strength. Unpublished theses, dissertations and other studies with null results warrant scrutiny for weaknesses, rather than automatic inclusion in a meta-analysis as unfortunate victims of “publication bias.” In light of the standing Coyne et al. (2011) recommendation, and other best practices standards, Carter et al.’s (2015) failure to provide methodological codes or otherwise assess the research quality of the student work included in their meta-analysis must be regarded as a serious deficiency that should not be emulated by future meta-analysts.

## Secondary Statistical Analysis and Interpretation of Results

Carter et al. (2015) conducted a series of secondary analyses to determine whether their *g* = 0.43 effect size estimate for depletion was exaggerated due to publication bias or other small study effects. Except for their last set of analyses using “Precision Effects” tests, described below, the Carter et al. (2015) analyses were consistent in demonstrating the existence of a non-zero effect in this data set. Such outcomes are more supportive of the resource depletion model than the null hypothesis.

The Test for Excess Significance (TES; Ioannidis and Trikalinos, 2007) examines whether there are more significant effects than should be expected based on the statistical power of the studies in the database. If the number of significant studies exceeds the expected value, then missing studies are presumed to be attributable to publication bias or other causes. Carter et al.’s (2015) estimate of power = 0.42 for the combined depletion studies (Table 5), for example, implies that only 50 of the 118 depletion effects (0.42 * 118) in the dataset should be statistically significant; more are “excess.” It also follows that if most of the 68 published effects in the present dataset are statistically significant [which may not be the case, but Carter et al.’s (2015) Table 1 lacks that information], the logic of TES suggests that the population could contain as many as 94 negative or non-significant depletion studies. Because the dataset contains 50 unpublished effects, most of which may be non-significant, up to 44 studies could be presumed by TES to be “missing” due to publication bias, despite the authors’ efforts to contact unpublished authors.

When TES was calculated based on the limits of the confidence intervals for the random-effects estimate, the range was seen as too large to be conclusive. Using random-effects meta-analysis estimates, no bias was suggested in four of the eight datasets (hand grip, possible anagrams, standardized tests, Stroop), but that possibility was raised about the other four tasks (food consumption, impossible puzzles, possible anagrams, and working memory). Yet, we question the wisdom of excluding most of the published literature, dividing the remainder into small categories, and then attempting to estimate if some significant effects are “excess” or some null studies are “missing.” The TES finding of no such problems in four of the depletion paradigms contradicts Carter et al.’s (2015) overall null conclusion.

Next, Carter et al. (2015) tried the Trim and Fill method, which attempts to estimate the impact of “missing” studies based on asymmetries in the distribution of obtained results (Duval and Tweedie, 2000; Moreno et al., 2009). The approach is based on the assumption that studies with low standard errors should approximate the true effect, and that studies with higher standard errors should be symmetrically distributed around the true effect. A greater number of confirming than disconfirming studies with moderate to high standard errors might be attributable to publication bias, with negative outcomes to seem to be “missing” and justifying the imputation of additional unsupportive data.

Carter et al.’s (2015) decision to feature eight depletion procedures might be defended because procedural subsamples are more homogenous than the full sample that includes diverse procedures — but their inclusion of methodologically questionable or deficient studies meant that the subsamples still had high heterogeneity. Trim and Fill is known to mistake heterogeneity for missing studies (Terrin et al., 2003). The use of Trim and Fill imputed no data in four of the eight outcomes (food consumption, working memory, impossible anagrams and standardized tests). For the remaining four data sets, between 1 and 5 additional “studies” were imputed, with even more added to the heterogeneous combined sample. Although the Trim and Fill estimate suggested the Stroop outcome is non-significant, the other seven procedures remained significant. Thus, even with the imputation of *k* = 29 hypothetical cases, the *g* across the 8 conditions was 0.24, *p* < 0.001, consistent with the validity of the resource depletion phenomenon in a variety of instantiations.

Then, Carter et al. (2015) used the Funnel Plot Asymmetry Test (FAT), which was again designed to test for small study effects by contrasting the number of studies that were and were not significant in relation to the statistical power (Egger et al., 1997). Carter et al. (2015) reported that there was no significant problem stemming from small study effects in five of the procedures: food consumption, impossible anagrams, possible anagrams, standardized tests, and working memory. Using the unusual standard of *p* < 0.10, the FAT flagged three of the same datasets that raised questions in Trim and Fill analysis (hand grip, impossible puzzles, Stroop). These findings confirm that small study effects are not a threat to the validity of most operationalizations of the resource depletion effect.

Although the depletion effect survived these tests, a question should be raised about the appropriateness of the TES, Trim-and-Fill and FAT in this context. Each test operates like an inferential statistic by requiring a sample that offers a valid estimate of the effect size, standard error, power and number of significant effects, from which conclusions about the literature may be derived. Yet, it is not clear which assumptions must be met in order for a sample to be appropriate for such inferences (cf. Ioannidis, 2013) or to what true population of studies a non-random sample of studies is presumed to refer. It seems that the more weak studies that are included in a meta-analytic sample, the lower the estimated effect size and statistical power, and the higher the estimated error, which leads TES, Trim-and-Fill and FAT to project the possibility of more studies in file drawers, even if such studies are non-existent.

It also should be reemphasized that statistical power is due, in large part, to the individual investigator’s methodological decisions about sample size and his or her skill in producing high impact and low error results. Statistical power and error can be estimated from a dataset, but they are not an intrinsic attribute of a procedure or a phenomenon. That is also true of a portion of effect size estimates, as our Conclusion will explain. For such reasons, Morey (2013) regards the use of tests like TES to detect and correct for publication bias and small study effects to be “questionable at best and completely misleading at worst (p. 182).” Even so, it should be reemphasized that the TES, Trim-and-Fill and FAT analyses largely left the resource depletion effects standing, rather than provided support for the null hypothesis.

## “Precision Effect” Tests Provide Inconclusive Results

After the depletion effect remained significant following several secondary analyses for publication bias and small study effects, Carter et al. (2015) employed two new and highly untested tests in questionable ways. The Precision Effect Test (PET; Stanley and Doucouliagos, 2014) models the relation of an estimated effect to the standard error. It uses the intercept of a weighted least squares (WLSs) regression model in which effect size estimates are regressed on the standard error of those estimates, weighted by the inverse of the variances. An alternate test from the same statisticians called Precision Effect Estimation with Standard Error (PEESE) employs the intercept from a similar model but uses variances instead of standard errors as the predictor. Thus, both methods use WLSs regression to look for relationships between effect sizes and errors (which are linked to sample sizes). Both methods attribute any obtained relationship between effect sizes and error to bias, and interpret the intercept of the regression model as an effect size estimate that has been corrected for bias. Those assumptions have not yet been strongly demonstrated or widely accepted.

The PET-PEESE procedures impose high penalties on studies with moderate to high standard errors. That can inappropriately reduce estimates of effect size when a high proportion of questionable or weak studies are included in the dataset. In addition, the accuracy and suitability of PET and PEESE for samples of less than 20 are unknown, because the PET-PEESE method was tested only on simulation data of sample sizes of 20 and 80. The developers of PET-PEESE expressed caution about its suitability for small samples: “The meta-regression sample size of 20 is…a rather small sample size for any regression estimate...regression-based estimators may not be appropriate if only a very small number of comparable empirical estimates exist (Stanley and Doucouliagos, 2014, p. 66).” After running their simulations, the test developers confirmed that: “These meta-regression estimates do not perform quite as strongly when there are only 20 estimates available (p. 71).” Because seven of the eight of Carter et al.’s (2015) depletion data sets have *k* < 20 studies, with a mean of *k* = 14.75, there are grounds to believe that results based on PET-PEESE with those samples are unreliable.

After removing a supportive study that they considered to be an outlier, Carter et al. (2015) report concerning their standardized test dataset that: “PET estimate (*b* = 0.60) and PEESE estimate (*b* = 0.46) of the depletion effect were both larger than the random-effects meta-analysis estimate (*g* = 0.30). In other words, the application of PET and PEESE to this data set actually provided increased estimates of the depletion effect (although these estimates were non-significant because WLS meta-regression models produce wider confidence intervals compared to random-effects meta-analysis) (p. 808).” So, PET and PEESE both indicate a medium effect size for strength depletion on standardized test performance. Other depletion outcomes were weaker, but the authors seem to encourage readers to accept the null hypothesis for depletion effects despite the fact that the PET-PEESE tests are unproven, have unusually wide confidence intervals, and were deployed on datasets that were as much as 40% smaller than those tested by the developers (without the readers being warned of that limitation). Such constraints seemed like undue obstacles for the depletion effect.

Carter et al. (2015) also made the surprising suggestion that their PEESE results indicate an *inverse* relation between self-control effort and performance effect sizes on four procedures. They suggested that the relation is “positive…for impossible anagrams, impossible puzzles, and working memory…and negative— contrary to the limited strength model …for food consumption, hand grip, possible anagrams, and Stroop (p. 809).” First, all PEESE results were non-significant (Table 7), so the signs of the coefficients should have been regarded as due to chance and not interpreted. Second, PET and PEESE do not test the relation of depletion to performance, but instead are secondary regression analyses that model the relation of the effect size’s variance or standard error to the magnitude of the effect size. Finally, unstable coefficients, including reversals of sign from the original correlations, are common problems when regression procedures are used on small or heterogeneous samples Small samples are less likely to meet statistical assumptions, such as normally distributed residuals. Indeed, Kerlinger and Pedhazur (1973) advised against regression analyses with less than 100 cases. Consequently, it is not surprising that artifacts emerged when Carter et al. (2015) used PET and PEESE on their eight extremely small data sets (i.e., *k* = 12 to 21), or their heterogeneous *k* = 118 samples.

Carter et al. (2015) were clear that: “We favor an interpretation of our findings that depends on the validity of the WLS meta-regression estimators PET and PEESE (p. 812).” Those analyses certainly offered the only basis for Carter et al. (2015) to claim that the ego depletion effect could be zero. Yet, additional serious deficiencies involving PET-PEESE recently have been reported by Inzlicht et al. (2016), using an extensive series of simulations, indicating that the procedures underestimate real effects and are prone to fail to find true differences, especially by PET and especially with heterogeneous datasets. In short, Carter et al. (2015) based their conclusions on new and untested statistical tests that might be ideal for research synthesists seeking to make nothing out of something — but were highly questionable and probably inappropriate if one was seeking to ascertain the reality behind the data.

## Conclusion

Carter and McCullough (2013a,b, 2014) have steadily argued against the strength depletion model, but Carter et al.’s (2015) latest conclusion, that the true depletion effect is zero, is untenable. Their own broadest analysis yielded a supportive *g* = 0.43 in favor of the model, which is not much smaller than what Hagger et al. (2010) found using only published studies. The 76% of the Carter et al. (2015) reports with a positive *g* for the depletion effect could be interpreted as persuasive evidence in support of the model.

To argue for a null effect, Carter et al. (2015) excluded 80% of the extant literature and most of the published studies, including the bulk of significant findings in the Hagger et al. (2010) meta-analysis. They also coded their studies inadequately; overemphasized unpublished studies by graduate students who produced null results; conducted and over-interpreted a dubious set of mini-meta-analyses; and tried a barrage of questionable, sometimes unsuitable, statistical assumptions, and procedures. Even so, only the unproven PET-PEESE offered null effects for seven of the eight depletion paradigms.

Carter et al.’s (2015) subtitle: “Self-Control Does Not Seem to Rely on a Limited Resource” conveys acceptance of the null hypothesis. Yet, Shadish et al. (2002) emphasized three criteria that should be met before the null hypothesis is accepted: (1) maximize statistical power (rather than minimizing it by including deficient studies and dividing studies into small categories with low power); (2) determine *a priori* what would count as meaningful effect (rather than relying on dichotomous significance testing); and (3) conduct a mixed effects meta-analysis that employs a range of thoughtfully chosen moderators to determine when effects can be found and when they cannot (rather than excluding many important moderators from the meta-analysis). Carter et al. (2015) argued for the acceptance of null conclusions while failing to meet any of those criteria.

One of the central aims of meta-analysis is to diminish the influence of researcher biases and increase transparency in order to estimate the true validity and strength of an effect. Unfortunately, the literature has taken on a tone that seems unduly suspicious both of the published literature and of investigator intentions. The unpleasant term “p-hacking” refers to a spectrum of research behaviors or choices that result in unwarranted significant findings (Simonsohn et al., 2014). Of course, the enthusiastic experimenter should not stop an experiment just when a significant result is found, conduct multiple statistical analyses in hopes that one will be significant, or conduct multiple studies until one is significant while withholding the non-significant studies from the report (Shadish et al., 2002). Now, however, research synthesists are encouraged to suspect that the literature is replete with Type I errors and conduct tests during meta-analyses to detect p-hacking, thereby implying researcher misconduct.

Yet, it is unreasonable to suggest that primary investigators are likely to be biased whereas research synthesists can safely be presumed to be impartial and immune from any confirmation bias. Instead, we suggest that the field should be equally concerned about both p-hacking and what might be called “p-bashing,” which creates Type II errors. P-bashing occurs when a skeptical research synthesist deliberately or unconsciously seeks to obtain small or non-significant effects sizes to overturn an established paradigm. This outcome can be obtained through, for example, the exclusion in a meta-analytic sample of a high proportion of successful studies, the inclusion of a high proportion of deficient studies, the use of questionable secondary analyses with unmet statistical assumptions that diminish effect size and the inclusion of secondary analysis of that reduce effect sizes while omitting secondary analyses that boost them (e.g., correction for attenuation, Hunter and Schmidt, 2004).

We do not know Carter et al.’s (2015) intentions and do not accuse them of p-bashing. On the contrary, we emphasize the broader point that the field’s approach to research results that are challenging to replicate should be mindful of the baby while throwing out the dirty bathwater. We suggest that methodological rigor and impartiality mandates comparable consideration and control of Type II errors as of Type I errors in both meta-analyses and replications; both p-bashing and p-hacking are incommensurate with scientific progress.

We suggest that a major goal of meta-analysts should be clarification of the moderators of when effects are reliably obtained, and when they are not. With one of the few moderators tested, Carter et al. (2015) found no advantage from being linked to the laboratories of Baumeister, Tice, and their students; other careful investigators produce effects of similar magnitude. Thoughtful readers might wonder why diverse researchers have conducted over 600 studies, and continue to do so, if there is no depletion effect. If there were 100s of methodologically valid but unsuccessful studies, their authors would not have been silent. Even before the widespread use of meta-analysis, the “invisible college” promptly recognized and abandoned wrong hypotheses and unworkable methods (cf. Rodin, 1981).

More broadly, the underlying assumption that there is a single “true” effect size for a phenomenon that is influenced by multiple variables, such as ego depletion, contains some elements of absurdity. Not only do the depletion manipulations, contexts and outcome measures vary considerably, but depletion itself occurs in varying degrees. In fact, some researchers have explicitly sought to compare mild vs. severe levels of depletion (e.g., Vohs et al., 2012), similar to the impact of varying degrees of physical tiredness (Evans et al., 2015). If we asked “How much more slowly does someone run a mile when tired than when fresh?” a thoughtful initial response should be more along the lines of “How tired?” rather than “precisely 20% slower.” The fact that meta-analyses found heterogeneous effect sizes for different depletion paradigms is not a weakness but instead is perfectly consistent with this analysis.

There doubtless is much else still to learn about ego depletion and self-control. Carter et al. (2015) might have made a positive contribution to the field by focusing on when and why some studies, manipulations and measures were better or worse for demonstrating the depletion effect. The most regrettable aspect of Carter et al.’s (2015) report is that it may discourage research on what may be a true effect, with the potential for important theoretical implications and practical applications. Our goal in pointing out the many questionable elements of the Carter et al. (2015) approach is to encourage continuing and ever-better thinking, research, and meta-analyses.

## Author Contribution

MC was the lead author and source of the new statistics. RB provided additional commentary, citations, and edits.

## Conflict of Interest Statement

The authors declare that the research was conducted in the absence of any commercial or financial relationships that could be construed as a potential conflict of interest. The reviewer NC and the handling Editor declared their shared affiliation, and the handling Editor states that the process nevertheless met the standards of a fair and objective review.

## Footnotes

1 omelet.

2 Dates, authorship and other information on the studies used in Carter et al. (2015) was drawn from a file posted at http://supp.apa.org/psycarticles/supplemental/xge0000083/xge0000083_supp.html.

3 Schmiechel published four studies from his dissertation. Carter et al. (2015) included them, plus a fifth study that he chose not to publish, so he has effects in both the published and unpublished groups.
